# Albendazolesulphoxide concentrations in plasma and hydatid cyst and prediction of parasitological and clinical outcomes in patients with liver hydatidosis caused by *Echinococcus granulosus*

**DOI:** 10.3325/cmj.2014.55.146

**Published:** 2014-04

**Authors:** Tomislava Skuhala, Vladimir Trkulja, Mislav Runje, Dalibor Vukelić, Boško Desnica

**Affiliations:** 1 University Hospital for Infectious Diseases “Fran Mihaljević,” Zagreb, Croatia; 2 Department of Pharmacology, Zagreb University School of Medicine, Zagreb, Croatia; 3 TAPI Research and Development Analytics, Pliva Croatia, Zagreb, Croatia

## Abstract

**Aim:**

To investigate the relationship between plasma and cyst concentrations of albendazolesulphoxide (ASO) and their effects on parasitological findings and disease recurrence in patients with liver hydatidosis.

**Methods:**

The study was conducted at the University Hospital for Infectious Diseases “Dr. Fran Mihaljević,” Zagreb, Croatia, between August 2006 and January 2011. Consecutive patients (N = 48, age 6-77 years) were treated with albendazole (3 × 5 mg/kg/d) over 28 days before surgical cyst removal (n = 34) or percutaneous evacuation (PAIR) (n = 14). Plasma ASO was determined on days 10 and 28 of treatment and cyst concentrations at surgery/PAIR.

**Results:**

Disease recurred in 3 surgically treated patients. Variability of ASO concentrations was substantial. Plasma concentrations on day 10 were higher than on day 28 (geometric means ratio [GMR] 2.00; 95%CI 1.38-2.91, *P* < 0.001) and higher than cyst concentrations at the time of treatment (GMR = 1.58, 1.01-2.34, *P* = 0.045). Higher cyst (but not plasma) concentrations were independently associated with lower odds of protoscolex motility (OR = 0.23, 0.01-0.70, *P* < 0.001) and higher odds of protoscolex destruction (OR = 1.17, 1.04-1.46, *P* < 0.001). With adjustment for age and protoscolex motility, higher day 10 plasma concentrations (but not cyst concentrations) were associated with lower odds of disease recurrence (OR = 0.49, 0.09-0.97, *P* = 0.035). Plasma concentrations did not predict cyst concentrations.

**Conclusion:**

Viability of protoscolices progressively decreased with increasing ASO concentrations in the cyst. Data strongly suggested that higher plasma concentrations reduced the risk of disease recurrence.

Echinococcosis or hydatid cyst disease is an anthropozoonosis caused by the larvae of *E. granulosus*, *E. multilocularis*, *E. vogeli,* and *E. oligarthus* (1). In transient hosts (eg, sheep, cattle, pigs, humans), the parasite develops in the form of hydatid cyst(s) of different sizes. The cyst consists of the outer layer (ectocyst), made of dense fibrous tissue; the middle layer (endocyst), which is elastic and lamellar in structure; and the inner (germinative) layer, which gives rise to buds that develop into scolices. The cyst content is hydatid fluid, produced by the germinative layer. In humans, the most commonly invaded organs are the liver and the lungs, but practically all organs can be affected (1-3). In Croatia, the disease is caused exclusively by *E. granulosus*. According to the European Hospital Morbidity Database (4) for the period 2010-2012, age-adjusted annual hospital admission rates due to echinococcosis (ICD-B67) in Croatia varied between 0.011 and 0.0167/1000 population (ie, between 51 and 86 cases/y), indicating that the diseases is relatively rare but still stably present.

Treatments for hydatid cyst disease include surgical removal of the cyst(s) (still most commonly used method); percutaneous aspiration of the cyst with instillation of a scolicidal agent (95% ethanol or hypertonic saline [15%-20%] or albendazole), ie, the PAIR (puncture, aspiration, instillation, and re-aspiration) procedure, which seems to have greater clinical efficacy and lower rate of complications than the surgical procedure; and treatment with benzimidazole anthelmintic drugs. The latter, pharmacological option might be used as a monotreatment in the case of smaller cysts or when invasive approaches are not feasible, but it is typically adjunctive treatment to surgery or PAIR used to prevent dissemination of scolices from ruptured cysts. In this setting, benzoimidazoles are used over at least 4 weeks before the definitive treatment (5-8). Among benzoimidazoles, albendazole is considered a cornerstone pharmacological treatment of echinococcosis. Although some controversies still exist regarding optimal dosing, the most widely accepted regimen implies administration of 10-15 mg/kg/d (5-8). Upon oral ingestion (with a fatty meal to increase bioavailability), albendazole is rapidly metabolized (first-pass metabolism in the liver) into the active form albendazolesulphoxide (ASO), which inhibits tubuline polymerization in the parasite microtubules and inactivates cell division (9). Greater systemic bioavailability is considered an important advantage of albendazole over mebendazole, the other member of the group (9). Although ASO has been shown to penetrate both the hepatic and non-hepatic cysts (9,10), the prognostic value of plasma and/or cyst concentrations has not been elucidated. Therefore, we aimed to relate ASO concentrations in the plasma and in the cysts, and to investigate their relationship with the parasitological and clinical outcomes in patients with liver hydatidosis treated with albendazole over one month prior to surgical treatment or PAIR.

## Patients and methods

### General design and ethics

This prospective observational prognostic study was conducted at a tertiary care university-affiliated teaching hospital between August 2006 and January 2011 and was approved by the institutional Ethics Committee. The only procedures that differed from the standard treatment/follow-up protocol were blood and cyst fluid samplings for determination of drug concentrations and laboratory evaluation of protoscolex viability.

### Patients

Eligible for inclusion were consecutive patients (all ages) with standard (5,8) clinical and radiological (ultrasonography [US] and computed tomography [CT]) criteria for liver echinococcosis with parasitologically proven disease (protoscolices in the cyst content samples) who met the following criteria: a) gave a written informed consent (guardians for children); and b) had a symptomatic or asymptomatic active echinococossis, ie, cyst types CL, CE1-CE3 according to the World Health Organization (WHO) classification system (11). The WHO classification is based on US images: (i) CL – unilocular cyst lesion with uniform anechoic content, round or oval, cyst wall not visible; (ii) CE1 and CE2 – active cysts. CE1 is unilocular simple cyst, round or oval with a visible wall, typically showing fine echoes due to shifting of brood capsules (“snow flake sign”), whereas CE2 is multilocular with daughter cysts; (iii) CE3 – anechoic cysts that are thought to be degenerating (transitional group). CE3a cysts feature the “water-lily” sign of floating membranes, whereas CE3b cysts are predominantly solid with daughter cysts; (iv) CE4 and CE5 are considered inactive. They are echogenic with increasing degrees of calcification and are nearly always nonviable. Patients with inactive disease (cyst types CE4 or CE5), patients with liver cysts <30 mm in diameter, pregnant women, patients treated with ritonavir or phenytoin, and patients with an active malignant disease were not included. The commenced albendazole treatment was to be stopped in patients showing hypersensitivity reactions to albendazole or developing other serious adverse events (particularly ≥5-fold increase in liver aminotransferases or granulocyte depletion to <0.5 × 10^3^/mm^3^).

### Study flow and procedures

**Figure 1:**
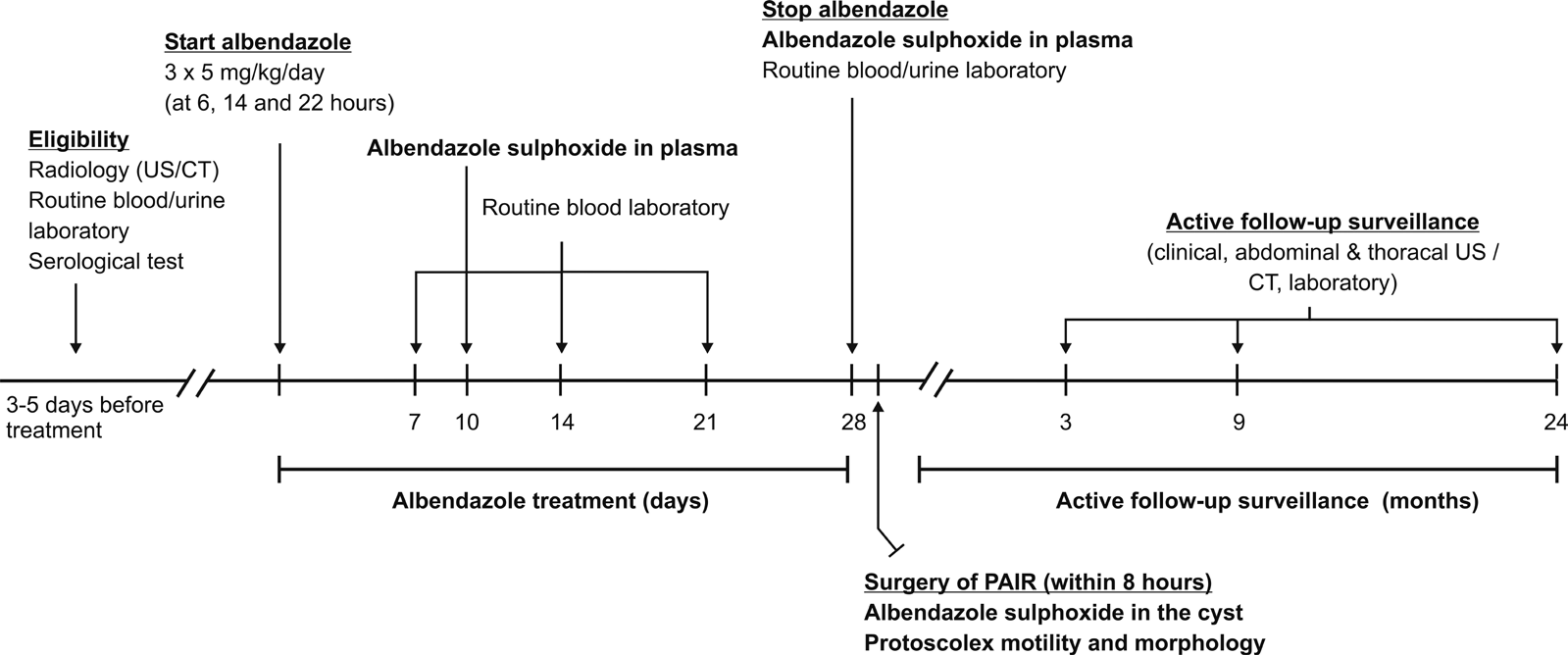
Study flow. Procedures of primary relevance are indicted in bold. US – ultrasound; CT – computed tomography; PAIR – percutaneous cyst evacuation (puncture, aspiration, instillation, re-aspiration).

Patients were hospitalized, routine biochemical/hematological tests and serological tests were performed, and 4-week treatment with albendazole was commenced (Figure 1). Albendazole (film-coated immediate-release tablets by Krka Pharmaceuticals (Novo Mesto, Slovenia) or Cilag-Janssen (Beerse, Belgium) 15 mg/kg/d was divided in three daily administrations (at 06:00, 14:00, and 22:00 hours) with a meal consisting of 20 g of fatty cheese(45% milk fat). Doses were calculated weekly and rounded to the nearest 100 mg. Routine biochemical/hematological tests were performed weekly and at the last day (day 28) of albendazole treatment. Patients were daily evaluated for potential albendazole side-effects. Peripheral venous blood samples (3 mL) for determination of plasma ASO concentrations were collected into heparinized Vacutainer tubes on days 10 and 28 of treatment at 09:00 hours, ie, at the time of the expected peak plasma concentration of ASO (3 hours after the first morning dose) (12). Plasma was separated and frozen at -72°C until assaying. Surgery or PAIR was performed on the day of the last albendazole dose (within 8 hours) (Figure 1). All surgeries (cystectomy or pericystectomy) were performed at the Department of Surgery University Hospital Center Zagreb. All PAIR procedures were performed at the University Hospital for Infectious Diseases “Fran Mihaljević,” Zagreb following the recommended WHO protocol (13) using 95% ethanol as a scolicidal agent. At surgery or PAIR, two samples of the cyst content were taken, one for parasitological evaluation and one (3 mL) for determination of ASO concentrations (frozen at -72°C until assayed) (Figure 1). Regular follow-up visits were scheduled at 3, 9, and 24 months after completion of treatment (Figure 1), when physical examination, routine biochemical/hematological laboratory tests, and ultrasound examination were undertaken. CT of the abdomen was performed in all patients at 9 and 24 months after treatment, and in those with suspected recurrence at 3 months after treatment. Chest CT was performed in all patients at the 24-month visit. Disease recurrence was defined as any of the following: a) appearance of a cyst at the evacuation site; b) evidence of a new cyst at a different liver site; or c) evidence of a new cyst in any other organ.

### Serodiagnostics

Sera were assessed for the presence of antibodies (IgG) against echinococcal antigens using a commercial semiquantitative enzyme-linked imunosorbent assay (ELISA) in line with the manufacturer’s instruction (the assay is based on purified hydatid liquid antigens [NovLisa^TM^, NovaTec GmbH, Leinfelden-Echterdingen, Germany]), and indirect fluorescence microscopy. For the latter analysis, after removal of fat using acetone, cryostat-madeprotoscolex slices were incubated (37°C for 30 minutes) with diluted sera (1:100 in phosphate buffer pH 7.2), rinsed (3 × 10 minutes), incubated (37°C, 30 minutes) with anti-human IgG antibodies labeled with fluorescin-izocyanate (Imunološki Zavod, Zagreb, Croatia), rinsed again, and analyzed (14). A patient was considered seropositive when ELISA titers were ≥1:100 and fluorescence microscopy titers were ≥1:10.

### Determination of albendazolesulphoxide concentrations

A validated high pressure liquid chromatography (HPLC) method with UV detection (wavelength 293 nm) developed by Hoaskey et al (15) and modified by Schipper et al (16) was used for determination of ASO concentrations in plasma and hydatid fluid. Briefly, after centrifugation (4000 rpm, 10 minutes, 4°C), the sample was extracted by acidic denaturation over 30 minutes (400 μL of 3% HCl added to 400 μmL of sample together with 20 mg/L of mebendazole hydroxide as internal standard) and supernatant separated by centrifugation at 14 000 rpm for 10 minutes. The HPLC parameters were as follows: Agilent 1200 system; 100 × 4.6 mm (3 μm) CN spherisorb column; injection volume 100 μL; flow rate 1.0 mL/min; mobile phase – 100 mmol acetic acid buffer with 15% methanol, pH 3.5; elution time for the internal standard 11 minutes; elution time for ASO 8 minutes. The lower limit of quantification (LLOQ) was 0.025 mg/L, linear range (r >0.999) was 0.025-4.00 mg/L, and recovery at the low (0.025mg/L, LLOQ), medium (1.021mg/L), and high (3.890mg/L) part of the range was 83.0%, 96.0%, and 88.0%, respectively. Inter-day precision (coefficient of variation) was 4.71% and intra-day precision was 7.28%.

### Parasitological evaluation

All evaluations were done by light microscopy at 37°C using unstained wet mount (17). Native specimens were first evaluated for the presence of protoscolices or their fragments to confirm the diagnosis of echinococcosis. Further analysis aimed to estimate the level of protoscolex viability based on motility and morphological characteristics. Analysis of protoscolex-rich sediment obtained by centrifugation (17) was deemed non-discriminative since virtually no motility was observed in any of the samples. Hence, to re-create conditions resembling those in the canine gastrointestinal system, 1 mL of each sample was incubated with 0.05 mL of undiluted canine bile or 0.2% solution of sodium taurocholate in 1.0 mL of balanced Hank’s buffer pH 7.2 for 48 hours at 37°C and it was analyzed after 24 and 48 hours of incubation (stimulation). Each sample was analyzed on at least 10 slides. Two outcomes were assessed: a) presence of any protoscolex showing motility (yes/no); b) proportion of observed protoscolices showing morphological signs of destruction(complete disintegration and free hooks, destruction of the tegmentum, loss off rostellar hook morphology, and loss of hooks) graded as “very good response” (>66%), “good response” (33%-66%), and “poor response” (<33%).

### Research questions and data analysis

We posed three primary research questions. 1. Pharmacokinetic: what is the relationship between plasma and cyst concentrations? Analyses were performed by fitting general linear (mixed) models to ln(c) data and the effects were expressed as geometric means ratios, GMR [GMR = exp〈ln(c_1_) - ln(c_2_)〉] or regression coefficients (β). Within-subject variability of plasma concentrations (day 10, day 28) was determined as relative standard deviation (%RSD = 100 x √e^s2wr^–1) 2. Parasitological: do plasma and/or cyst ASO concentrations affect parasitological outcomes? Proportions of patients with motile protoscolices/patients with morphologically changed protoscolices were analyzed by fitting generalized linear models and results were expressed as odds ratios (OR). 3. Clinical outcome: are ASO concentrations related to disease recurrence? This relationship was investigated by generalized linear models (effects expressed as OR). We used SAS 9.3 statistical software (SAS Inc., Cary, NC, USA) licensed to Zagreb University School of Medicine.

## Results

### Patient characteristics and description of outcomes

**Figure 2:**
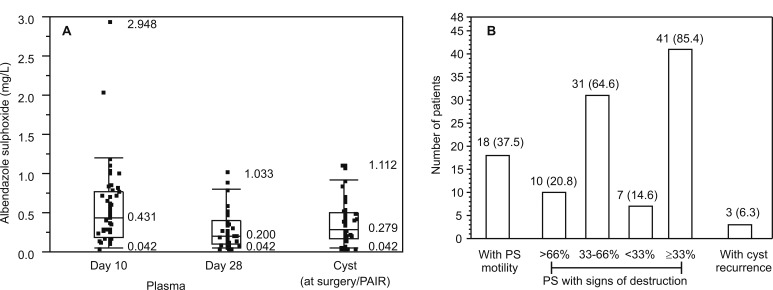
Study outcomes. (**A)** Pharmacokinetic outcomes: albendazolesulphoxide concentrations in plasma (days 10 and 28 of treatment) and in the cyst (obtained at surgery or PAIR). Shown are individual values (N = 48), medians, quartiles and inner fences (lower = 1st quartile – 1.5 × interquartile range; upper = 3rd quartile +1.5 × interquartile range). Values falling “out of the fences” are outliers. Numerical values are median, minimum, and maximum. (**B**) Parasitological and clinical outcomes: number (%) of patients with signs of protoscolex (PS) motility or with different proportions of PS showing signs of destruction, and patients with disease recurrence. PAIR – percutaneous evacuation of the cyst (puncture, aspiration, instillation, re-aspiration).

A total of 48 patients were enrolled, 34 (70.8%) submitted to surgery and 14 to PAIR as the final treatment. Diagnosis was confirmed parasitologically in all enrolled patients. Their characteristics are summarized in Table 1. No patient developed hypersensitivity to albendazole. No apparent trend in hematological/biochemical laboratory tests during treatment was observed apart from normalization of the initially high erythrocyte sedimentation rate and C-reactive protein values (not shown). Transient eosinophilia was noted in 6 patients (12.5%) and elevations of liver enzymes, but not reaching the critical values, in another 6. Hence, all 48 patients completed albendazole and final treatments and the 24-month follow-up. ASO concentrations in plasma were higher on day 10 than on day 28 and than concentrations in the cyst (Figure 2A). The range of individual patient values was huge (25 to 80-fold differences between minimum and maximum) (Figure 2A). Signs of protoscolex motility were observed in 18/48 patients; ”good” morphological response (33%-66% PS show signs of destruction) was observed in 31/48 patients, and “good or very good” morphological response (≥33% PS show signs of destruction) was observed in 41/48 patients.

**Table 1:** Patient characteristics*

Demographics:	
age (years)	53.5 (6-77) (2 children 6 and 7 y)
men	21 (44.0)
**Epidemiological and clinical particulars:**	
positive epidemiological anamnesis	43 (89.6)
clinically symptomatic	28 (58.3)
first/recurrent episode	37 (77.1) / 11
serologically positive	31 (67.4)
US cyst classification: CL/CE1/CE2/CE3^†^	4 (8.3)/15 (32.3)/16 (33.3)/13 (27.1)
fertile cyst (CL or CE1 or CE2)	35 (72.9)
**Laboratory (blood):**	
leukocytes ( × 10^9^/L)	6.4 (3.5-13.7)
eosinophils (fraction)	0.020 (0-0.180)
erythrocyte sedimentation rate (mm/3.6 ks)	15 (1-80)
C-reactive protein (mg/L)	2.5 (0.2-81.7)
aspartate aminotransferase (U/L)	22 (12-179)
alanineaminotransferase (U/L)	22 (9-481)
gama glutamyltransferase (U/L)	26 (7-281)
alkaline phosphatase (U/L)	69.5 (30-215)
**Final treatment:**	
surgery/PAIR^‡^	34 (70.8) (26 pericystectomy, 8 cystectomy)/ 14

*Data are presented as median (range) or n (%).

†WHO classification of the cysts based on ultrasound (US) characteristics (1). Types CL, CE1 and CE2 are “fertile cysts,” whereas type CE3 shows signs of involution.

‡PAIR – percutaneous cyst evacuation (puncture, aspiration, instillation, re-aspiration).

### Relationship between ASO concentrations in plasma and the cyst

**Figure 3:**
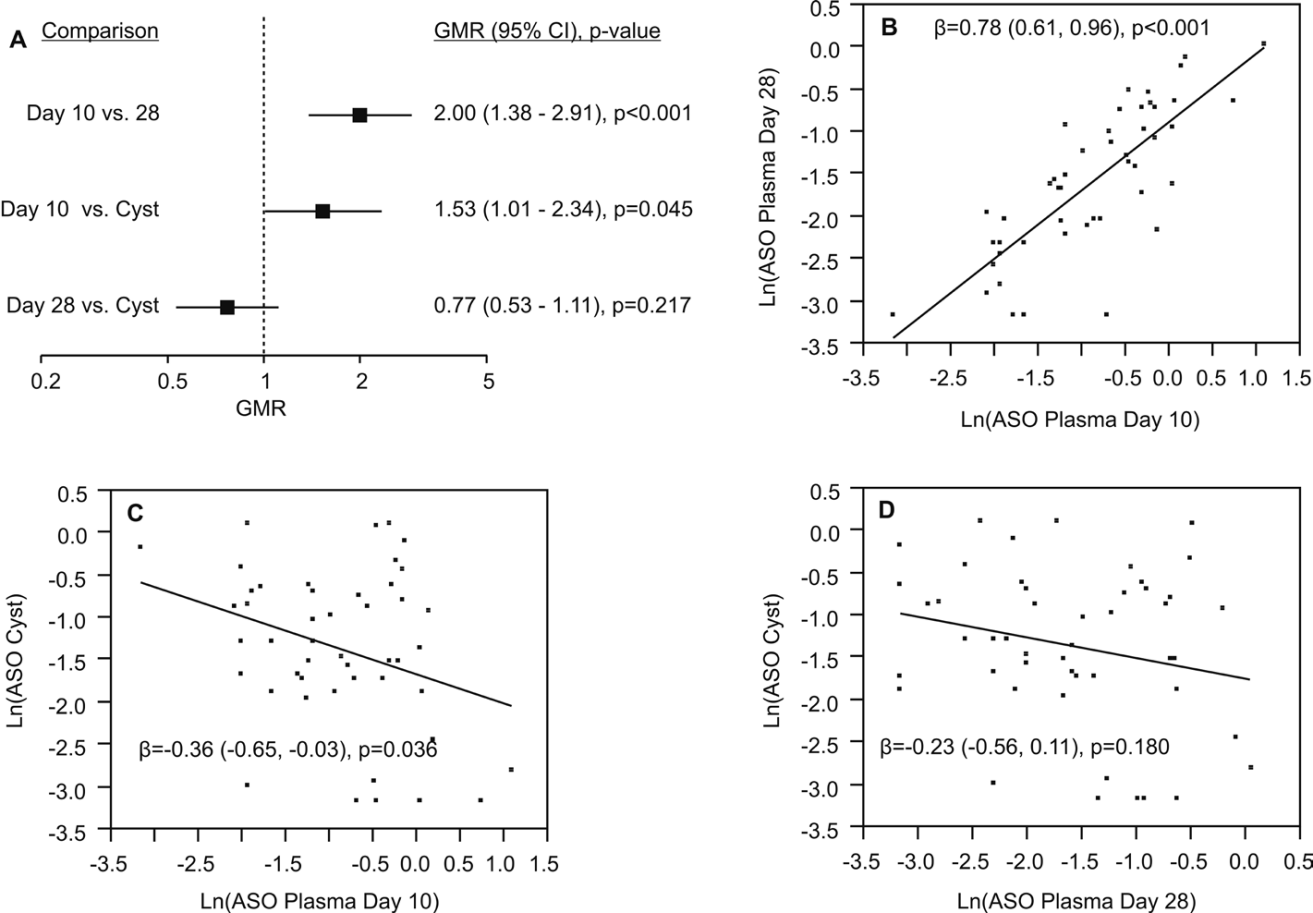
Relationship between albendazolesulphoxide (ASO) concentrations in plasma and in the cyst. (**A**) Differences between ASO concentrations. Plasma and cyst ln(concentrations) were considered as repeated-measures data and were analyzed in a general linear mixed model accounting for autocorrelation and with adjustment for age, sex, and cyst classification (fertile [CL or CE1 or CE2] vs CE3). Differences are expressed as geometric means ratios (GMR) with 95% confidence interval (CI). (**B**) Relationship between ln(plasma concentrations) on day 10 and day 28. Day 28 values were a dependent variable. Depicted is regression coefficient (β) with confidence intervals for day 10 concentrations as a predictor, adjusted for age, sex, and cyst classification (fertile vs CE3). (**C**) Relationship between day 10 ln(plasma concentrations) and ln(cyst concentrations). Cyst values were a dependent variable. Depicted is the adjusted (as above) effect (β) of day 10 concentrations. (**D)** Relationship between day 28 ln(plasma concentrations) and ln(cyst concentrations). Cyst values were a dependent variable. Depicted is the adjusted (as above) effect (β) of day 28 concentrations.

None of the factors depicted in Table 1 was associated with plasma or cyst ASO concentrations (not shown). Plasma concentrations on day 10 were twice higher (*P* < 0.001) than on day 28 (Figure 3A) and around 53% higher than in the cyst (at surgery/PAIR) (*P* = 0.046) (Figure 3A). Within-subject variability of plasma concentrations was very high (RSD = 105%). Plasma concentrations taken on day 28 were around 23% (but not significantly) lower than cyst concentrations (Figure 3A). Higher plasma concentrations on day 10 were independently associated with higher concentrations on day 28 (Figure 3B). They were, however, associated with lower cyst concentrations, albeit weakly (Figure 3C). No association between plasma ASO concentrations taken on day 28 and cyst concentrations was observed (Figure 3D). There was no difference in plasma or cyst ASO concentrations between patients with fertile (CL, CE1, CE2, n = 35) and involutive (CE3, n = 13) cysts (not shown).

### Relationship between plasma and cyst ASO concentrations and parasitological outcomes

**Figure 4:**
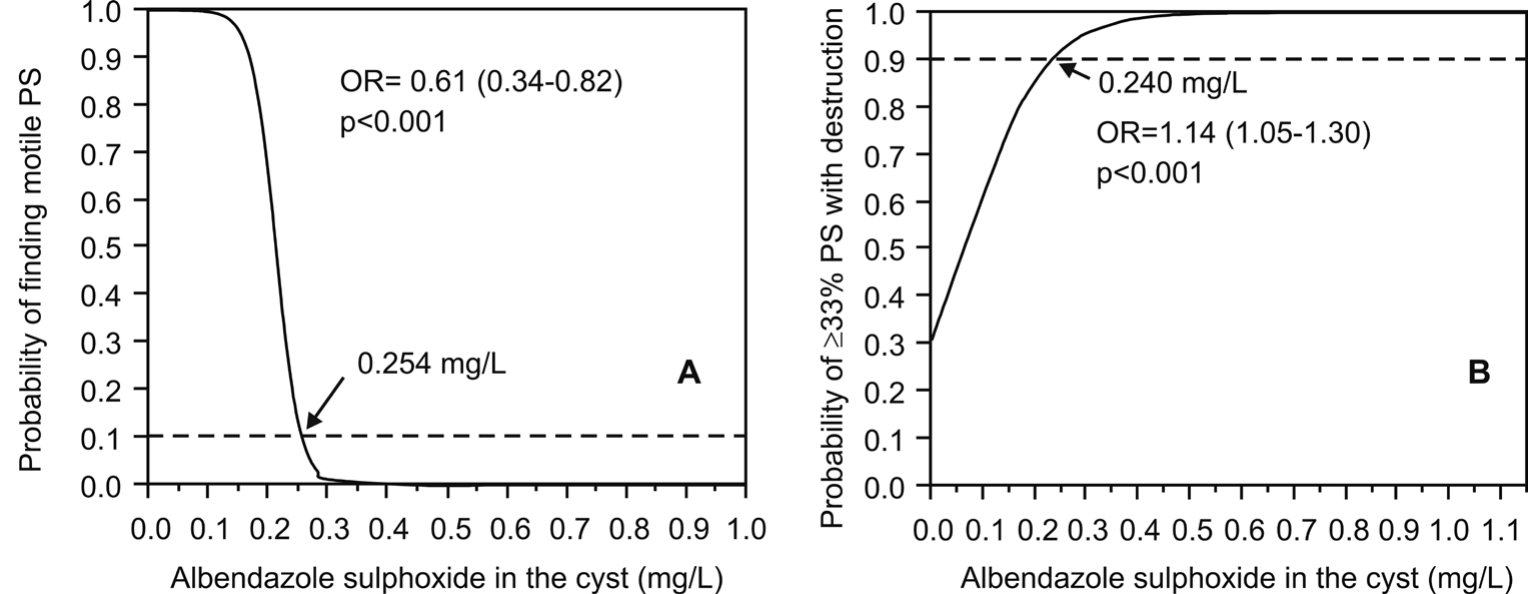
Univariate association between cyst albendazolesulphoxide concentrations and parasitological outcomes: (**A**) proportion of patients with signs of protoscolex (PS) motility and (**B**) proportion of patients with ≥33% of PS showing signs of destruction (“good or very good” morphological response). Estimated probabilities are from logit models with albendazolesulphoxide concentrations in the cyst as the only independent variable. Depicted are odds ratios (OR) with 95% confidence intervals by 0.01 mg/L increase in drug concentration. Arrows indicate concentrations at which probabilities fall to ≤10% (**A**), ie, exceed 90% (**B**).

For all patients, parasitological findings were identical after 24 hours and after 48 hours of incubation with canine bile/sodium taurocholate. Data for 48 hours are presented. Cyst ASO concentrations were strongly univariately associated with parasitological outcomes: 0.01 mg/L increase in concentration was associated with a 39% reduction in odds of finding signs of protoscolex motility (*P* < 0.001) (Figure 4A) and with 14% increase in odds of having ≥33% protoscolices with signs of destruction (“good or very good” morphological response) (*P* < 0.001) (Figure 4B). Cyst ASO concentrations ≥0.250mg/L were associated with ≤10% probability of finding motile protoscolices and with ≥90% probability of finding “good or very good” morphological response (Figure 4). Plasma ASO concentrations determined on day 10 showed a weak univariate association (*P* = 0.070) with protoscolex motility, which, however, was abolished with adjustment for cyst concentrations (not shown). No other trend of univariate association between plasma concentrations and parasitological outcomes was observed. None of the factors depicted in Table 1 showed even a trend (*P* < 0.250) toward univariate association with either parasitological outcome (not shown). With adjustment for age, sex, “fertile cyst,” and obtaining of sample during surgery or PAIR, ASO cyst concentrations (by 0.01 mg/L) were independently associated with lower odds of motile protoscolices (OR = 0.23, 95%CI 0.01-0.70, *P* < 0.001) and higher odds of “good or very good” morphological response (OR = 1.17, 95% CI 1.04-1.46, *P* < 0.001).

### Relationship between plasma and cyst ASO concentrations and disease recurrence

The three patients with disease recurrence were all women, treated surgically (2 pericystectomy, 1 cystectomy), all with previous (before the index) disease episodes and all tested seropositive at the start of the index treatment. Their characteristics were: case 1) age 66 years, CL (fertile) cyst, ASO plasma concentrations 0.149 and 0.132 mg/L on days 10 and 28, respectively, and cyst concentration 0.502 mg/L. Parasitological findings indicated no signs of protoscolex motility and >66% protoscolices with signs of destruction; case 2) age 42 years, CE1 (fertile) cyst, ASO plasma concentrations 0.290 and 0.188 mg/L on days 10 and 28, respectively, and cyst concentration 0.222 mg/L. Parasitological finding indicated protoscolex motility, and 33%-66% protoscolices with signs of destruction; case 3) age 72 years, CE3 (signs of involution) cyst, ASO plasma concentrations 0.188 and 0.042 mg/L on days 10 and 28, respectively, and cyst concentration 0.155 mg/L. Parasitological findings indicated no signs of protoscolex motility and <33% protoscolices with signs of destruction. With adjustment for age and presence of protoscolex motility, higher day 10 plasma ASO concentrations were associated with around 50% lower odds of disease recurrence considering all (*P* = 0.035) or only surgically treated patients (*P* = 0.041) (Table 2). Day 28 concentrations showed a trend toward univariate (Table 2), but not independent association (not shown), whereas cyst concentrations were unrelated to disease recurrence (Table 2).

**Table 2:** Association between albendazolesulphoxide (ASO) concentrations and disease recurrence*

	All patients (N = 48)		Surgically treated (N = 34)	
	odds ratio (95% confidence interval)	*P*-value	odds ratio (95% confidence interval)	*P*-value
**Univariate**				
ASO plasma, day 10 (by 0.1 mg/L)	0.58 (0.17-1.01)	0.061	0.62 (0.19-1.04)	0.084
ASO plasma, day 28 (by 0.1 mg/L)	0.47 (0.07-1.12)	0.113	0.52 (0.08-1.17)	0.147
ASO cyst (by 0.1 mg/L)	0.89 (0.47-1.33)	0.622	0.89 (0.47-1.33)	0.604
**Multivariate^†^**				
ASO plasma, day 10 (by 0.1 mg/L)	0.49 (0.09-0.97)	0.035	0.50 (0.09-0.98)	0.041
Age (by 5 y)	1.61 (0.93-4.04)	0.102	1.89 (0.97-5.87)	0.065
No signs of protoscolex motility	0.35 (0.01-10.9)	0.500	0.26 (0.00-9.82)	0.442

*Logit models were fit to proportion of patients with recurrence considering all patients and only those treated surgically (since all 3 cases were treated surgically). Odds ratios are with profile-likelihood 95% confidence intervals and *P*-values are from likelihood-ratio tests.

**†**Plasma concentrations, age, signs of motility, protoscolex morphology, cyst type and previous disease episodes were entered into model and then successively removed if *P* > 0.500.

## Discussion

The present study emphasizes the importance of attaining higher concentrations of the active albendazole metabolite, ASO, in the hydatid cyst in order to achieve a more pronounced protoscolex inactivation in patients with liver hydatidosis. The study further suggests two important conclusions: a) that cyst concentrations might not be predictable from the plasma concentrations; and b) that neither ASO concentrations in the cyst nor the level of protoscolex viability (at the end of a 4-week albendazole treatment) are predictive for a long-term clinical outcome. Rather, data suggest that higher plasma ASO concentrations (reflecting the tissue concentrations) are associated with a reduced risk or disease recurrence. Introduction of albendazole in treatment of hydatid cyst disease enabled a more liberal surgical approach and development of percutaneous cyst evacuation techniques by reducing the risks associated with spillage of the cyst content (5-8). Attempts are made to develop newer formulations allowing higher albendazole’s bioavailability (12) and optimization of treatment regimens, although some controversies still exist (5-8). Relationships between drug (ie, active metabolite, ASO) concentrations in plasma and the cyst and parasitological and clinical outcomes are important issues in this setting. The present study aimed to contribute to this matter.

The limited number of patients was due to a relatively low disease incidence and our intention not to exceed a 1.5-year recruitment period in order to maintain consistency of study procedures. In this respect, and considering that the disease relapsed in only 3 patients during the study follow-up, the present observations relating higher plasma ASO concentrations to a reduced risk of disease recurrence should be taken with caution. It should be noted, however, that our intention was not to detect “all possible factors” that might have influenced any of the analyzed outcomes, but to test the pre-defined hypotheses about ASO concentrations while accounting for (likely) the most relevant confounders. In line with this, the fact that we found no relationship between patient or disease characteristics (eg, age, sex, seropositivity, symptomatic diseases, cyst type, pre-treatment laboratory findings) with plasma or cyst ASO concentrations, parasitological outcomes, or disease recurrence does not “disqualify” these factors as potentially relevant – the study was simply not designed to formally test their effects. At the same time, this did not compromise our observations. On the other hand, lack of drop-outs, high adherence to the procedures, the use of a validated method of ASO quantification, and a standardized parasitological evaluation enabled a fair level of internal validity. Indirectly, present observations were externally validated by the fact that the observed ASO cyst concentrations were within the range of values reported in other studies with a similar dosing schedule, which, however, were conducted in considerably fewer patients (10,18,19). The fact that the study was conducted in a “regular practice” setting emphasizes its relevance.

With the limitation of being based on only two single-point determinations (both at the expected time of peak concentrations, C_max_, after the first morning dose), the observations related to plasma ASO concentrations were in agreement with the known high inter- and intra-subject variability. The fact that the values on day 10 of treatment were higher than on day 28 (end of treatment) could be due to several reasons. In a pharmacokinetic study in healthy volunteers (20), total exposure to ASO was reduced, elimination half-life shortened, and clearance was increased after 15 days of repeated dosing of albendazole. Since volume of distribution was unchanged, data indicated time-dependent kinetics of ASO, likely due to induction of metabolizing enzymes (20). However, healthy volunteers do not have a hydatid cyst. Combined with the fact that C_max_ in healthy participants was not affected by repeated dosing (20), the presently observed higher ASO concentrations in the cyst determined within 8 hours since the last dose than in plasma at the time of morning C_max_ on day 28, suggest that the observed day 10 to day 28 change could have also been due to a gradual distribution of ASO into the cyst. Data clearly demonstrate the importance of higher cyst concentrations for reduction of protoscolex viability. The lack of association between plasma concentrations and parasitological findings is in line with the observed lack of their association with concentrations in the cyst. Still, present data strongly suggest that higher plasma concentrations are associated with a lower risk of disease recurrence. Although this observation might appear paradoxical (considering the lack of the effect on protoscolex viability), it seems pharmacodynamically plausible. The risk of disease recurrence after surgical cyst removal/PAIR is largely attributed to contamination due to the spillage of the cyst content. Clearly, the ability of shed protoscolices to generate a new cyst will depend on their viability. However, tissue ASO concentrations are also relevant since they could inhibit otherwise viable protoscolices. Animal studies show that ASO is readily distributed into different tissues where attained concentrations are comparable to plasma concentrations (eg, heart, lungs, spleen, colon) or are two (eg, kidney, small intestine) to four times higher (liver) (21). Once cyst has been removed, it appears reasonable to consider tissue ASO concentrations, reflected by the plasma concentrations, as an important factor in determination of the risk of disease recurrence. Our findings support the need for further studies investigating the relationship between more thoroughly evaluated systemic exposure to ASO (eg, repeated daily plasma concentration-time profiles or cumulative total exposure based on several daily concentrations) and disease recurrence as the primary clinical endpoint.

In conclusion, the present analysis in patients with liver hydatidosis treated with albendazole 15 mg/kg/d over 28 days before surgery or PAIR demonstrates the importance of attaining higher ASO concentrations in the cyst for reduction of protoscolex viability. Cyst concentrations were not predictable from plasma concentrations determined at the expected morning C_max_ on days 10 and 28 of treatment. However, this does not exclude the possibility that a more detailed plasma concentration-time profiling would be predictive of the cyst ASO concentrations. At the same time, the study strongly suggests that higher plasma concentrations are associated with a lower risk of disease recurrence.
